# Unlocking collective resilience: the mediating role of team behaviors in the relationship between the structure and performance of sports startups

**DOI:** 10.3389/fpsyg.2026.1790379

**Published:** 2026-03-25

**Authors:** Yan-Ting Wang, Zong-Nan Liu, Guo-Liang Lin, Juan Du

**Affiliations:** 1 School of Physical Education, Quanzhou Normal University, Quanzhou, China; 2 Faculty of Education, The National University of Malaysia, Bangi, Selangor, Malaysia; 3 Faculty of Education & Liberal Sciences, City University Malaysia, Petaling Jaya, Malaysia; 4 Department of Public Physical Education, Fujian Agriculture and Forestry University, Fuzhou, China

**Keywords:** IPO framework, resilience-relevant behaviors, sports startups, team behavior, team performance, team structure

## Abstract

Given the high uncertainty and resource constraints faced by sports startups, it is critical to explore the impact of internal team mechanisms on venture performance. While extensive research has addressed leadership, cohesion, and performance in professional sports teams and coaching staff, systematic quantitative analyses of sports *entrepreneurial* teams remain scarce. To address this gap, this study integrates the input–process–output (IPO) framework with the concept of resilience-relevant behaviors to construct and test an integrated “structure–behavior–performance” model. The direct and indirect effects of structural dimensions on multidimensional performance via specific behavioral processes are examined systematically, clarifying how resilience-relevant behavioral shapes these pathways in resource-scarce sports entrepreneurship contexts. A quantitative research design was employed. Data were collected through a survey of 250 core team members from 97 sports startups in Quanzhou and Zhenjiang, China. Confirmatory factor analysis (CFA) and structural equation modeling (SEM) were conducted using SPSS 22.0 and AMOS 24.0 to test the hypothesized paths and mediating effects. The results indicate that (1) team structure significantly shapes team behavior: A clear role structure promotes learning and decision-making behaviors, a rational power structure enhances decision-making and risk-taking behaviors, and skill structure significantly improves risk-taking behavior, although its impact on learning behavior is not significant. (2) Team behaviors differentially drive performance: Decision-making behavior comprehensively enhances cohesion, explicit performance, and developmental performance; learning behavior primarily increases cohesion and explicit performance; and risk-taking behavior significantly positively affects developmental performance. (3) Mediating mechanisms are confirmed: Team behaviors serve as significant mediators between team structure and performance, validating the efficacy of optimizing structural inputs to trigger specific behavioral processes that enhance multidimensional outcomes. This study reveals how different dimensions of team structure translate into short-term explicit performance and long-term dynamic capabilities, providing theoretical evidence and practical guidance for team governance in sports startups.

## 1 Introduction

In the context of rapid technological iteration and evolving consumer demands, sports startups have increasingly become pivotal drivers of economic vitality and social development. Compared with individual entrepreneurship, team-based entrepreneurship is associated with higher survival rates and success probabilities through complementary roles, collective decision-making, and resource integration, particularly in multidisciplinary collaboration and high-uncertainty environments (Jin et al., 2017; Cardella et al., 2021). Operating across diverse sectors, such as sports, health, technology, and commerce, the inherent complexity and uncertainty of sports startups impose rigorous demands on team structural design and behavioral operations (Ratten, 2011; Pei et al., 2020; Cardella et al., 2021).

Although the literature has extensively highlighted the significance of leadership, cohesion, and athletic performance within professional sports teams, coaching staff, and event organizations (Carron et al., 2002; Cotterill, 2012), systematic quantitative examinations of sports entrepreneurial teams remain insufficient—specifically regarding the causal pathways and mediating mechanisms within the “structure–behavior–performance” nexus (Jin et al., 2017; Horvatinovic et al., 2023). Previous research has seldom examined how team structure translates into performance outcomes through specific behavioral processes, nor has it fully explored the evolutionary mechanisms under conditions of high uncertainty and resource constraints. This knowledge gap is particularly pronounced in the field of sports entrepreneurship. Teams must simultaneously adapt to market volatility, regulatory shifts, and evolving consumer expectations regarding health and innovation—encompassing stakeholder management (athletes, fitness enthusiasts, sports organizations, and regulators) and balancing commercial outcomes with sport-specific metrics (e.g., user engagement, athletic performance enhancement, and health outcomes). Yet empirical research on how structural configurations facilitate behavioral adaptation under these sport-specific pressures remains limited. This knowledge gap constrains both the theoretical depth and managerial guidance available to sports startups.

The operational logic of sports startups is highly distinctive, distinguishing them from general technology or manufacturing ventures. First, sports startups pursue hybrid performance goals that extend beyond financial returns to include sporting legitimacy, reputation, and social value creation (Ratten, 2011; Cardella et al., 2021). This necessitates a more balanced power structure (PS) within the team to navigate competing commercial and social demands (Neckebrouck and Zellweger, 2024). Second, sports markets exhibit high event-driven volatility and seasonality, where demand is often tied to competition cycles and athlete performance (Wills et al., 2023; Appelqvist et al., 2016). Such uncertainty places immense pressure on learning (LB) and decision-making behaviors (DMB) as teams strive for strategic responsiveness (Wiese et al., 2021). Third, these ventures are deeply embedded in local communities and institutional networks, relying on relational governance with sports associations and fans rather than purely market-based exchanges (Misener and Doherty, 2013). Finally, sports entrepreneurial teams often feature hybrid identity configurations, where members simultaneously act as athletes, coaches, and managers (Ratten, 2011). This identity hybridity intensifies coordination challenges, making the clarity of role structures (RS) and the activation of resilience-relevant behaviors particularly salient in determining venture success (Boyd et al., 2021). Consequently, examining team mechanisms within this context is not merely an application of general theory but a necessary extension that accounts for the unique institutional and market logics of the sports industry.

Accordingly, this study integrates the input–process–output (IPO) framework with the concept of resilience-relevant behaviors to construct and test an integrated “structure–behavior–performance” model. The core variables are defined as follows: at the input level, the study focuses on a three-dimensional team structure—role structure (RS), skill structure (SS), and power structure (PS); at the process level, three categories of team behaviors serve as mediating variables—learning behavior (LB), decision-making behavior (DMB), and risk-taking behavior (RTB); and at the output level, the study targets three performance dimensions—developmental performance (DP), team cohesion (TC), and explicit performance (EP). This research systematically examines the direct and indirect effects of structural dimensions on multidimensional performance through specific behavioral processes. Simultaneously, embedding a resilience perspective into the IPO framework helps explain the heterogeneity of structure–behavior–performance pathways within the volatile and resource-scarce environments characteristic of sports entrepreneurship (Su and Junge, 2023; Allal-Chérif et al., 2023). The research focus is categorized into three levels: direct pathways (direct effects of structure on performance), behavioral mediating pathways (the role of behaviors as channels between structure and performance), and the moderating or augmenting role of resilience-relevant behavioral (how resilience-relevant behaviors or stabilizes these pathways, particularly across different performance dimensions).

Sports startups navigate numerous challenges within the rapidly evolving sports market—such as technological disruption in fitness, wearables, and athlete management platforms—alongside seasonal revenue fluctuations and resource constraints (Pellegrini et al., 2020; Qi et al., 2024; Seçkin et al., 2023; Yu and Wang, 2021). Their members must learn quickly, coordinate decisions, and embrace risk-taking to sustain and enhance performance amid adversity (Wiese et al., 2021; Miao et al., 2017). In this turbulent environment, we view these team-level processes—learning, decision-making, and risk-taking—as collective manifestations of employee resilience. Unlike individual psychological traits, collective employee resilience stems from dynamic interactions among team members (Stoverink et al., 2018; Bowers et al., 2017). It represents the overall capacity of employees to absorb shocks and reorganize resources through shared behavioral mechanisms, rather than functioning as an independent resilience variable (Duchek, 2019; Su and Junge, 2023). Particularly in sports entrepreneurship, these adaptive behaviors must balance unique demands: commercial sustainability alongside sport-specific performance goals, stakeholder expectations, and health/social impact objectives (Pellegrini et al., 2020). Behavioral resilience offers a systematic analytical lens for integrating individual and team capacities for coping, recovery, and reorganization (Su and Junge, 2023; Duchek, 2019). It reveals how teams in resource-constrained entrepreneurial settings enhance dynamic capabilities, cohesion, and observable performance through adaptive learning, collaborative decision-making, and controlled risk exploration (Yu and Wang, 2021; Wiese et al., 2021; Miao et al., 2017). By focusing on resilience-related behavioral processes, this research advances theoretical development at the intersection of team effectiveness and organizational resilience. It avoids treating resilience as an isolated behavioral construct, thereby maintaining conceptual clarity. Simultaneously, it offers valuable insights for sports startups seeking to build high-performing teams under conditions of uncertainty.

## 2 Literature review

### 2.1 Input–process–output framework

The IPO model, originally proposed by McGrath (1964), serves as a foundational framework for explaining how teams transform initial resources into performance outcomes through internal interaction mechanisms. According to this model, inputs encompass team structure, individual characteristics, and contextual factors, which define the boundary conditions for team operations. Processes refer to the behavioral and psychological interactions among team members during collaboration—such as communication, learning, risk-taking, and decision-making—acting as the critical link between inputs and outputs. Outputs are manifested as task performance, team cohesion, innovation capability, and long-term developmental potential (Marks et al., 2001; Ilgen et al., 2005).

Notably, the IPO framework is particularly well-suited to illuminate the dynamics of sports startups, which operate at the intersection of commercial entrepreneurship and sports-specific performance requirements. Unlike general ventures, sports startups must simultaneously optimize for business sustainability (e.g., revenue growth, market expansion) and sports-related outcomes (e.g., athlete performance improvement, user engagement in fitness platforms, health impact). This dual-outcome imperative means that team structural choices and behavioral adaptations in sports entrepreneurship are uniquely constrained and multifaceted, making systematic analysis through the IPO lens both theoretically compelling and practically essential.

In the fields of entrepreneurship and sport management, the IPO framework has been widely adopted to explain how startup teams survive and thrive in resource-constrained and highly uncertain environments. For instance, in project-based and high-risk industries, the IPO model has been utilized to reveal how structural characteristics reduce errors and enhance efficiency through behavioral processes (Manser, 2009; Tabassi et al., 2017). In entrepreneurial contexts, research emphasizes how team structural diversity and resilience-relevant behaviors assist new ventures in gaining competitive advantages (Clarysse and Moray, 2002; Fletcher and Sarkar, 2013). Similarly, in sports teams and event organizations, studies have shown that the IPO model helps elucidate how effective coordination, collective decision-making, and shared leadership transform diverse inputs into high-performance outputs (Cotterill, 2012; Svensson et al., 2019).

However, a critical distinction emerges when applying the IPO framework to sports startups as distinct organizational entities. While existing IPO applications in sports research predominantly focus on well-established athletic teams, coaching structures, or event management (Salcinovic et al., 2022), systematic examination of sports entrepreneurial teams—which face compounded pressures of startup resource scarcity, market uncertainty, and sports-specific stakeholder complexity (athletes, fitness, communities, regulatory bodies, sports organizations)—remains sparse (Pellegrini et al., 2020; Pedras et al., 2019). Sports startups operate under unique contextual demands: (1) volatile market conditions driven by rapid technological innovation (e.g., wearables, AI-driven training platforms) and shifting consumer preferences in sports and fitness (Ferreira et al., 2021); (2) multi-stakeholder alignment requirements that span commercial viability and sports integrity (Pedras et al., 2019; Svensson, 2017); and (3) the necessity to integrate general entrepreneurial competencies with sports domain expertise (Pellegrini et al., 2020). These characteristics necessitate a refined IPO analysis that explicitly accounts for the sports entrepreneurial context, rather than simply adopting general entrepreneurship or professional sports frameworks (Mathieu et al., 2017; Pellegrini et al., 2020).

#### 2.1.1 Team structure

Team structure is widely recognized as one of the fundamental input elements that shape team processes and outcomes. A substantial body of research indicates that structural factors—such as role clarity, skill diversity, and power distribution—significantly influence team collaboration, decision-making, and performance (Gladstein, 1984; Bunderson and Sutcliffe, 2003). However, a unified classification standard for team structural dimensions has yet to emerge in academia. For example, Stewart (2006) emphasizes the importance of task allocation and communication channels, while Park (2011) highlights power distribution and role interdependencies.

With respect to specific dimensions, role structure can reduce ambiguity and conflict, facilitating smoother collaboration and thereby improving team efficiency (Ucbasaran et al., 2003; Methot et al., 2015). In sports startups specifically, clear role delineation is particularly critical because teams must integrate roles spanning sports domain expertise (e.g., athlete experience, sports science knowledge), technology/innovation capabilities (e.g., product development, platform management), and business operations (e.g., commercial strategy, stakeholder management)—a multidisciplinary integration rarely found in traditional ventures (Ratten, 2011).

Skill structure emphasizes the diversity and complementarity of knowledge and expertise, which contributes to enhanced innovation and adaptability (Beckman et al., 2006; Aven and Hillmann, 2017). For sports startups, skill complementarity becomes even more salient, as teams require simultaneous expertise in sports science, technology innovation, regulatory compliance (e.g., athlete safety, data governance), and market understanding—creating a high bar for team assembly and integration (Pei et al., 2020). This multi-competency requirement distinguishes sports startups from general technology or service ventures, where domain expertise may be less critical.

Power structure reflects the distribution of authority and decision-making rights within the team; research suggests that balanced or shared leadership mechanisms can foster higher-quality decision-making and stronger team cohesion (Pearce and Conger, 2003; Svensson et al., 2019). In sports entrepreneurship, power distribution is complicated by the presence of domain experts (e.g., former athletes or coaches) who may possess high legitimacy in sports contexts but limited business experience, alongside business-oriented founders with limited sports credibility. Thus, designing power structures that leverage both sources of expertise while maintaining decision-making agility becomes a distinctive structural challenge in sports startups (Horvatinovic et al., 2023). Drawing on these studies, this research defines team structure through three core dimensions—role structure, skill structure, and power structure—and anticipates that they significantly influence subsequent team behaviors (Mathieu et al., 2018; Carter et al., 2018; Greer et al., 2018).

#### 2.1.2 Team behavior

Team behavior refers to the interaction patterns and adaptive practices exhibited by team members to accomplish tasks, solve problems, and respond to environmental challenges. Existing research generally emphasizes that behavioral processes represent the “black box” connecting structural inputs to performance outcomes (Marks et al., 2001). Within the framework of organizational resilience, this study specifically frames these behaviors as ‘Collective Employee Resilience.’ Drawing on the view that resilience is a dynamic process rather than a static asset (Williams et al., 2017), we argue that when employees in sports startups face structural constraints and market uncertainty, their collective resilience is enacted through specific behavioral patterns. Team learning, decision-making, and risk-taking thus serve as the functional mechanisms through which employee resilience is mobilized to sustain venture performance. In the contexts of entrepreneurship and sports organizations, learning, decision-making, and risk-taking behaviors are commonly regarded as the core of team effectiveness (Hmieleski and Ensley, 2007; Fletcher and Sarkar, 2013).

Specifically, learning behavior manifests as the proactive acquisition and sharing of knowledge, which not only enhances innovation capabilities but also strengthens team resilience (Edmondson, 1999; Zahra et al., 2008). Decision-making behavior represents how a team integrates information to reach collective judgments; studies have shown that decision-making processes that balance inclusiveness and decisiveness contribute to improved efficiency and cohesion (Eisenhardt, 1989; Hoeber and Hoeber, 2012). Risk-taking behavior reflects a team’s willingness to take action in pursuit of opportunities under uncertain conditions, which is particularly crucial for entrepreneurial and sports teams operating in highly volatile environments (Simons and Peterson, 2000; Núñez-Pomar et al., 2020). In this study, learning, decision-making, and risk-taking behaviors are treated as core team behaviors, serving as mediating processes that translate structural characteristics into performance outcomes.

#### 2.1.3 Team performance

Team performance is the ultimate output in the IPO model, encompassing both quantifiable task outcomes and indicators of team relationships and adaptability. Previous studies have generally categorized performance into dimensions such as explicit performance, developmental potential, and relational outcomes (Ensley et al., 2002; Kozlowski and Ilgen, 2006), although the emphasis varies across contexts. For instance, Lumpkin and Dess (1996) primarily measure performance through financial and market results, whereas Carron et al. (2002) highlight the central role of cohesion in sports teams.

Developmental performance reflects a team’s ability to acquire resources, drive innovation, and maintain long-term adaptability, which is key to sustainable growth (Teece, 2007; Eisenhardt and Martin, 2017). Cohesion reflects the emotional and task-based bonds among team members; empirical evidence shows that it is a significant predictor of effectiveness and persistence in entrepreneurial and sports teams (Ensley et al., 2002; Beal et al., 2003). Explicit performance serves as the most direct indicator of success, such as profitability, sales growth, and customer expansion, directly determining the market competitiveness of new ventures (Zahra and Covin, 1995; Hoch, 2012). In this study, team performance is defined in three dimensions—developmental performance, cohesion, and explicit performance—allowing a multidimensional assessment of sports startup team effectiveness.

In conclusion, the IPO model provides a robust theoretical framework for analyzing the team operations of sports startups. Role, skill, and power structures serve as input conditions, defining task division, professional complementarity, and authority distribution, respectively (Bunderson and Sutcliffe, 2002; Ensley et al., 2006). Learning, decision-making, and risk-taking act as process variables, embodying the “resilience practices” of teams in uncertain contexts (Edmondson, 1999; Hmieleski and Ensley, 2007). Finally, these behavioral processes impact output results such as developmental performance, cohesion, and explicit performance (Kozlowski and Ilgen, 2006; Teece, 2007; Zahra et al., 2008). Consequently, the IPO framework not only reveals the direct relationships between structure, behavior, and performance but also provides a logical explanation for exploring the mediating mechanisms of behavior within this nexus.

### 2.2 Hypothesis development

The selection of the three structural dimensions (role, skill, and power structure) is grounded in classical team design theory, which highlights task division, resource configuration, and authority distribution as the core organizing principles of collective action (Mathieu et al., 2018; Carter et al., 2018; Greer et al., 2018). Similarly, learning, decision-making, and risk-taking behaviors represent three fundamental behavioral domains in entrepreneurial settings: knowledge integration, strategic choice, and opportunity exploration (Lattacher and Wdowiak, 2020; Camuffo et al., 2019; Li and Ahlstrom, 2019; Dias et al., 2019). Together, these dimensions capture the most theoretically salient pathways through which teams translate structural conditions into multidimensional performance (Mathieu et al., 2018; Carter et al., 2018).

On the basis of the input-process-output (IPO) framework, this study conceptualizes team structure as the input dimension, team behavior as the process dimension, and team performance as the output dimension. Team structure, characterized by role structure (RS), skill structure (SS), and power structure (PS), shapes the modes of team interaction, which activates the collective resilience of employees manifested as learning behavior (LB), decision-making behavior (DMB), and risk-taking behavior (RTB). These behaviors subsequently impact team cohesion (TC), explicit performance (EP), and developmental performance (DP).

On this basis, the following hypothetical framework is proposed:

#### 2.2.1 Team structure and team behavior

##### 2.2.1.1 Role structure and team behavior

Role structure reflects the clarity of task division and functional responsibilities among team members (Bunderson and Sutcliffe, 2002). Prior research suggests that clearly defined roles reduce conflicts, enhance coordination, and promote learning (Edmondson, 1999). In entrepreneurial contexts, Ucbasaran et al. (2003) found that role differentiation improves decision-making effectiveness. Similarly, Ensley et al. (2006) reported that appropriate role allocation fosters collaboration and strategic execution. On this basis, it is proposed that role structure positively influences learning and decision-making behaviors.

*H1a*. Role structure positively affects learning behavior (RS → LB).

*H1b*. Role structure positively affects decision-making behavior (RS → DMB).

##### 2.2.1.2 Skill structure and team behavior

Skill structure refers to the diversity and complementarity of members’ expertise and knowledge (Bunderson and Sutcliffe, 2002). Teams with diverse skills are more innovative and adaptive, as members can complement each other’s deficiencies and broaden the team’s knowledge base (Barney, 1991; Beckman et al., 2006). Research further indicates that skill heterogeneity enhances exploratory and risk-taking tendencies (Cardella et al., 2021). Therefore, it is hypothesized that skill structure facilitates both learning and risk-taking behaviors.

*H2a*. Skill structure positively affects learning behavior (SS → LB).

*H2b*. Skill structure positively affects risk-taking behavior (SS → RTB).

##### 2.2.1.3 Power structure and team behavior

Power structure concerns the distribution of authority and influence in teams (Cogliser and Schriesheim, 2000). Previous studies have emphasized that power allocation significantly affects decision quality and team outcomes. For instance, Pearce and Conger (2003) argued that shared leadership improves collective judgment, while Eisenhardt and Bourgeois (1988) showed that authority distribution influences risk orientation. Svensson et al. (2019) also found that balanced power enhances team innovation. Therefore, power structure is expected to strengthen decision-making and risk-taking behaviors.

*H3a*. Power structure positively affects decision-making behavior (PS → DMB).

*H3b*. Power structure positively affects risk-taking behavior (PS → RTB).

#### 2.2.2 Team behavior and team performance

##### 2.2.2.1 Learning behavior and team performance

Learning behavior refers to the process through which team members actively acquire, share, and apply knowledge (Edmondson, 1999). Active learning is not only a means of knowledge accumulation but also a manifestation of members’ adaptability in dynamic and uncertain environments, enabling teams to remain flexible and continuously improve under external pressures. Empirical studies have shown that learning behavior enhances team cohesion and collective resilience (Beal et al., 2003), and through strengthened communication and knowledge sharing, it gradually fosters a culture of resilience characterized by trust, collaboration, and adaptive capacity. Such a culture of resilience emphasizes that teams exhibit positive coping behaviors through learning and adaptation when facing adversity (Fletcher and Sarkar, 2013), thereby improving cohesion and the likelihood of long-term survival.

Moreover, learning behavior not only strengthens both emotional and task cohesion but also improves explicit performance outcomes such as efficiency and product quality (Zahra et al., 2008). More importantly, sustained learning contributes to knowledge accumulation and experience, building dynamic capabilities that foster long-term development and innovation (Teece, 2007). Accordingly, the following hypotheses are proposed:

*H4a*. Learning behavior positively affects team cohesion (LB → TC).

*H4b*. Learning behavior positively affects explicit performance (LB → EP).

*H4c*. Learning behavior positively affects developmental performance (LB → DP).

##### 2.2.2.2 Decision-making behavior and team performance

Decision-making behavior captures the collective process of problem identification, option evaluation, and execution (Cohen and Bailey, 1997). Research suggests that effective decision-making strengthens member alignment and cohesion (Hoeber and Hoeber, 2012), directly contributes to organizational performance outcomes (Shen et al., 2021), and supports long-term adaptability and development (Ott et al., 2017). Thus, the following hypothesis is proposed:

*H5a*. Decision-making behavior positively affects team cohesion (DMB → TC).

*H5b*. Decision-making behavior positively affects explicit performance (DMB → EP).

*H5c*. Decision-making behavior positively affects developmental performance (DMB → DP).

##### 2.2.2.3 Risk-taking behavior and team performance

Risk-taking behavior refers to a team’s willingness to embrace uncertainty and pursue opportunities despite potential failure (Hmieleski and Ensley, 2007). In entrepreneurial settings, risk-taking has been identified as a key driver of innovation and long-term growth (Núñez-Pomar et al., 2020; Coombes and Nicholson, 2021). Therefore, the following hypothesis is proposed:

*H6*. Risk-taking behavior positively affects developmental performance (RTB → DP).

#### 2.2.3 Mediating role of team behaviors

##### 2.2.3.1 Learning behavior as a mediator

Role structure has been linked to enhanced knowledge exchange and collaboration (Beckman et al., 2006). Through stimulating learning behavior, role clarity not only fosters trust and cohesion among members (Beal et al., 2003) but also improves efficiency and explicit outcomes (Zahra et al., 2008). Thus, learning behavior is hypothesized to mediate the effects of role structure on team outcomes.

*H7a*. Learning behavior mediates the relationship between role structure and team cohesion (RS → LB → TC).

*H7b*. Learning behavior mediates the relationship between role structure and explicit performance (RS → LB → EP).

##### 2.2.3.2 Decision-making behavior as a mediator

Power structure shapes the quality of decision-making by influencing authority and participation (Eisenhardt and Bourgeois, 1988). Effective decision processes enhance adaptability and strategic choices, which in turn promote developmental performance (Teece, 2007). Hence,

*H8*. Decision-making behavior mediates the relationship between power structure and developmental performance (PS → DMB → DP).

##### 2.2.3.3 Risk-taking behavior as a mediator

**Figure 1:**
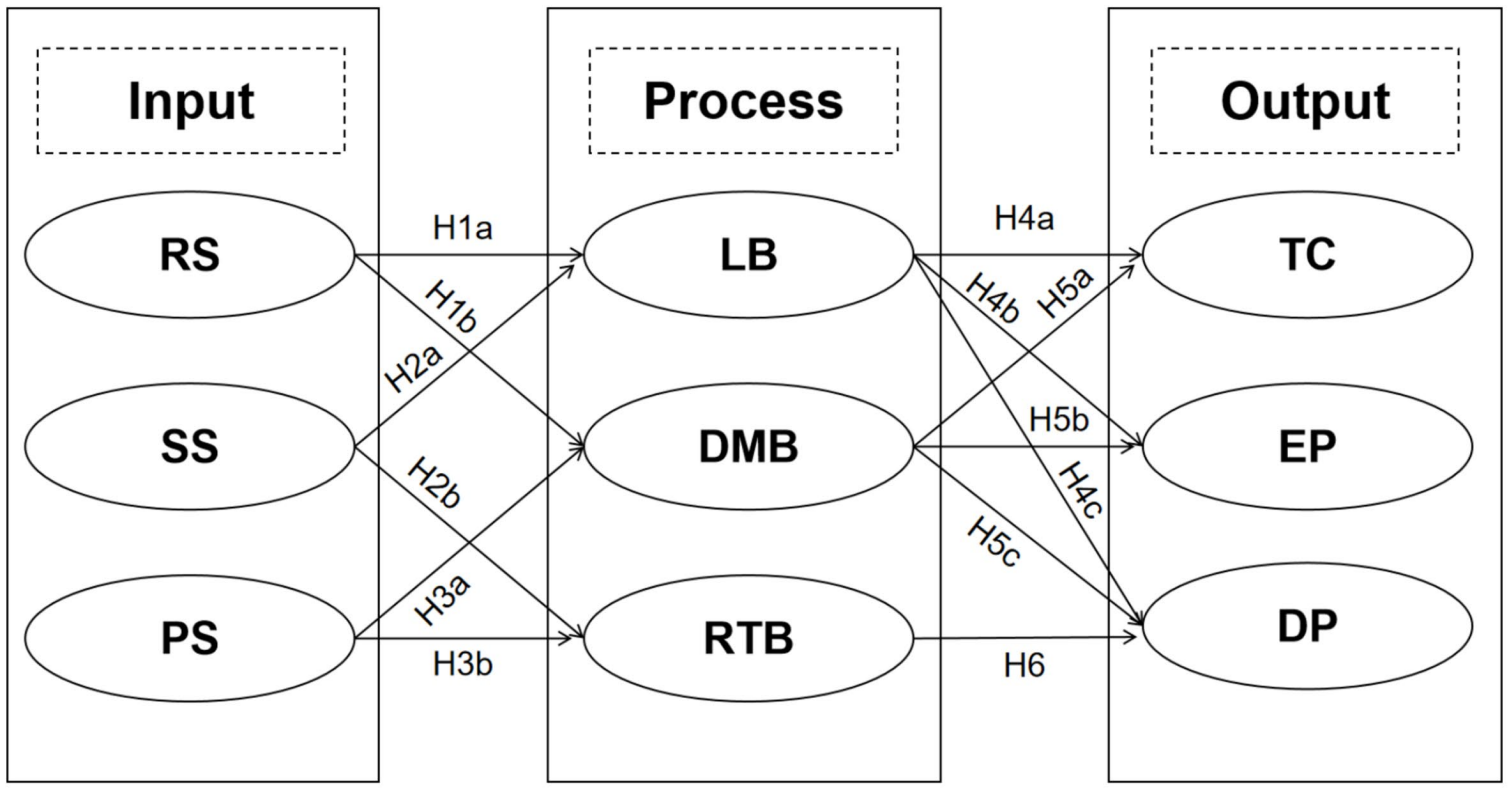
Proposed model. Indirect effect (mediating): H7a: RS → LB → TC; H7b: RS → LB → EP; H8: PS → DMB → DP; H9: SS → RTB → DP. Source(s): Authors’ own work.

Skill diversity expands cognitive resources and strengthens the willingness to take risks (Cardella et al., 2021). Such risk-taking acts as a resilience practice (Aven and Hillmann, 2017), enabling teams to adapt and grow in uncertain environments (Figure 1). Therefore,

*H9*. Risk-taking behavior mediates the relationship between skill structure and developmental performance (SS → RTB → DP).

## 3 Methodology

This study aims to investigate the impact of team structure and team behavior on the performance of entrepreneurial teams within sports startups. A quantitative research design was selected on the basis of the following considerations: First, quantitative methods allow the effective collection of large-scale sample data, thereby enhancing the research coverage and the reliability of the conclusions (Polit and Beck, 2010). This approach generates quantifiable and replicable data, facilitating the use of numerical language to elucidate the associations between team structure, team behavior, and team performance (Sandelowski, 2000), which ensures the statistical significance and objectivity of the findings (Davies, 2003). Second, quantitative data aid in the generalizability of the research outcomes, providing valid references for entrepreneurial teams in sports startups across various sectors (Stockemer, 2018).

### 3.1 Research measures

The data collection instrument consisted of two primary modules. The first section presents, the respondents’ demographic information, including gender, age, position, years of work experience, monthly income, and educational background. The second section focuses on nine theoretical dimensions: power structure, role structure, skill structure, risk-taking behavior, decision-making behavior, learning behavior, developmental performance, cohesion, and explicit performance.

All dimensions were measured using validated multi-item scales adapted from previous studies. Each item was rated on a five-point Likert scale (ranging from 1 = “Strongly Disagree” to 5 = “Strongly Agree”). Composite scores for each dimension were calculated and subsequently analyzed using structural equation modeling (SEM).

#### 3.1.1 Team structure

The questionnaire for entrepreneurial team structure comprised nine items, drawing on classic research on team structure. It was designed across three dimensions—role structure, skill structure, and power structure—referencing the classification of team structural elements by Xie et al. (2010).

Role Structure (RS): Drawing on the functional division and configuration principles of key roles (e.g., technical, marketing, and management experts) developed by Shane et al. (1995) and Ucbasaran et al. (2003), three items were designed. A sample item is “On my entrepreneurial team, suitable individuals play key roles such as technical, marketing, management, financial, and PR experts.”

Skill Structure (SS): Based on the importance of skill diversity and complementarity emphasized by Bunderson and Sutcliffe (2002) and Beckman et al. (2006), three items were developed. A sample item is “On my entrepreneurial team, the skills among members are largely complementary.”

Power Structure (PS): Based on the research on power concentration and distribution by Cogliser and Schriesheim (2000) and Pearce and Conger (2003), three items were formulated. A sample item is: “On my entrepreneurial team, the distribution of power is scientific, ensuring a basic alignment between authority and responsibility.”

#### 3.1.2 Team behavior

A nine-item questionnaire was designed to measure three dimensions of entrepreneurial team behavior: learning, decision-making, and risk-taking.

Risk-Taking Behavior (RTB): Items were adapted from Miller and Friesen (1982) regarding the commitment of substantial risky resources, combined with Gupta and Govindarajan’s (1984) views on risk tolerance and ambiguity and Covin and Slevin’s (1989) focus on proactive opportunity-seeking in uncertain environments. Sample items include “Team members do not blame each other even if a decision fails to meet expectations” and “Team members are willing to proactively seek ways to mitigate operational risks”.

Decision-Making Behavior (DMB): The items were adapted primarily from the strategic decision-making mode scale developed by Zhou and Gu (2008). Sample items include “Before major decisions are made, expert opinions and brainstorming ideas are weighed and accepted” and “Major decisions respect both democratic discussion and voting mechanisms”.

Learning Behavior (LB): Items referenced Van den Hooff and Van Weenen’s (2004) research on proactive and passive knowledge sharing and the “collaborative-aggressive” scale by Chen and Hao (2008). Key items include “I am willing to proactively share the new knowledge required for decision-making” and “My entrepreneurial team remains sensitive to dynamic changes in the external environment”.

#### 3.1.3 Team performance

The entrepreneurial team performance scale included three dimensions—developmental performance, cohesion, and explicit performance—for a total of nine items.

Developmental Performance (DP): Items referenced Chen and Chang, (2009) collective innovation scales, as well as Lumpkin and Dess (1996) and Zahra and George’s (2002) work on entrepreneurial orientation and absorptive capacity. Sample items include “Our entrepreneurial team prioritizes the accumulation of experience, knowledge, opportunities, and resources more than our competitors” and “Innovation supports the sustainable development of our team”.

Cohesion (TC): Items referenced Wang’s (2005) team cohesion scale. Key items include “Team members trust each other and frequently interact and collaborate” and “Team members can tolerate each other’s mistakes without following others blindly”.

Explicit Performance (EP): This evaluates quantifiable outcomes such as growth and profitability, drawing on Ensley et al. (2006) and Delmar and Shane (2004). Items include “Compared with competitors, our profitability is very strong” and “Compared with competitors, our sales growth is very rapid.”

### 3.2 Data collection

In accordance with the established definition of entrepreneurial teams, the participants in this study were selected on the basis of the following criteria: original idea proponents, equity investors, technology shareholders, key decision-makers with a direct influence on strategic direction, and senior executives committed to the creation and management of the entrepreneurial team. A convenience sampling method was employed, targeting 97 sports startups located in Quanzhou, Fujian Province, and Zhenjiang, Jiangsu Province, China. The selection criteria for these enterprises included (1) an establishment period of no more than 5 years, (2) a core business focused on the sports industry, and (3) a team size ranging from 3 to 20 members. These two regions were selected because of their vibrant sports entrepreneurship ecosystems and diverse enterprise types, which facilitated the acquisition of primary data. The researchers initially contacted the primary heads of the startups to obtain a list of team members from those willing to participate, after which the team members were mobilized for the survey.

The data were collected from October 2024 to March 2025. Given the geographical distribution of the subjects, onsite questionnaire distribution was the primary method utilized. A total of 270 questionnaires were distributed to the entrepreneurial teams of 97 sports startups, including Shengyan Tennis Club, Naldo Football Club, Aishi Tennis Club, and Minjiang Sports Development Co., Ltd. Of these, 250 valid responses were recovered, yielding an effective response rate of 92.59%. According to the general principles proposed by Babbie (2013), a recovery rate of 50% is considered adequate; thus, the response rate in this study meets the academic requirements.

### 3.3 Data analysis

In this study, a comprehensive analysis of the participants’ demographic characteristics, including gender, age, monthly income, educational background, and years of work experience, was conducted. The final measurement model included 27 observed indicators across all latent constructs. With a sample size of 250, the ratio of sample size to observed variables was approximately 9.26:1. According to commonly accepted SEM guidelines recommending a minimum ratio of 5:1 to 10:1, the current sample size is considered adequate for CFA and SEM analyses (Deng et al., 2018; Jak et al., 2020; Babbie, 2013). Data analysis was performed using SPSS 22.0 and AMOS 24.0 software. Exploratory factor analysis (EFA) and confirmatory factor analysis (CFA) were employed to assess the scale’s structure, while Cronbach’s alpha and variable correlation analysis were used to verify the reliability and validity of the measures. Furthermore, the goodness-of-fit of the structural model, as well as the testing of direct and mediating hypotheses, were conducted using AMOS 24.0.

### 3.4 Ethical considerations

This study was conducted in strict accordance with ethical standards. Prior to data collection, the research protocol was reviewed and approved by the Institutional Review Board (IRB) of [Quanzhou Normal University] under approval number [QZSYLL202525]. All participants were fully informed of the study’s objectives, the voluntary nature of their participation, and their right to withdraw at any time without incurring any liability. Informed consent was obtained from all the respondents prior to their involvement. Data were collected anonymously and used solely for academic research purposes, ensuring the privacy and information security of all participants.

## 4 Results

### 4.1 Participants’ demographics

This study presents the demographic characteristics of the participants. Of the total sample, 74% were male and 26% were female. The age of the participants was primarily concentrated in the 26–30 year age group (44.4%). With respect to educational background, 44% of the participants (*n* = 110) held a bachelor’s degree. In terms of monthly income, 54.8% (*n* = 137) earned between 5,001 and 10,000 RMB, while 10.0% (*n* = 25) earned more than 15,001 RMB. With respect to work experience, the majority of the participants (51.6%, *n* = 129) had 3 to 6 years of experience (including the 3rd year).

Although the demographic profile of the sample is presented in Table 1 to ensure transparency, these variables were included as control variables in the Structural Equation Modeling analysis. Their inclusion aimed to partial out variance attributable to individual differences. Preliminary tests indicated that these demographic factors did not exert a significant confounding effect on the primary hypothesized relationships, thereby supporting the robustness of the structural model.

**Table 1:** Participants’ demographics (*n* = 250).

Variable	Category	Frequency	Percentage (%)
Gender	Male	185	74.0
	Female	65	26.0
Age group	18–25	31	12.4
	26–30	111	44.4
	31–40	79	31.6
	41–50	28	11.2
	51–60	1	0.4
	Over 60	0	0
Work experience	1–3 years (inclusive)	6	2.4
	3–6 years (inclusive)	61	24.4
	6–10 years (inclusive)	129	51.6
	10 years and above	44	17.6
	10 years and above	10	4.0
Monthly salary	5,000 RMB and below	11	4.4
	5,001–10,000 RMB	137	54.8
	10,001–15,000 RMB	77	30.8
	15,001–20,000 RMB	14	5.6
	20,001 RMB and above	11	4.4
Education	Junior high school and below	2	0.8
	High school/Technical secondary school	52	20.8
	Junior college	84	33.6
	Bachelor’s degree	110	44.0
	Graduate degree	2	0.8

### 4.2 Measure validation

In this study, exploratory factor analysis (EFA) was conducted to evaluate the reliability and dimensional characteristics of the measurement scales, with the cut-off criteria for factor extraction set at an eigenvalue > 1.0. Principal component analysis (PCA) with varimax orthogonal rotation confirmed the structural dimensionality of the scales. The results indicated that the total variance explained reached 75.983%, exceeding the recommended threshold of 50%. The Kaiser–Olkin (KMO) coefficient was 0.860, surpassing the critical value of 0.70, which is statistically significant (Bartlett’s test of sphericity: *p* < 0.001; *χ*^2^ = 3307.935, df = 351), thereby fully supporting the suitability of factor analysis. The factor loadings for all the items of the latent variables were significant (ranging from 0.654 to 0.865), consistently exceeding the standard threshold of 0.50 (Hair et al., 2019), indicating that the scales possess good construct validity. Reliability testing revealed that the Cronbach’s alpha coefficients ranged from 0.782 (power structure) to 0.878 (risk-taking behavior), all of which were higher than the critical reliability standard of 0.7 (Nunnally and Bernstein, 1994; Hair et al., 2019). Reliability testing revealed that the Cronbach’s alpha coefficients ranged from 0.782 (power structure) to 0.878 (risk-taking behavior), all of which were higher than the critical reliability standard of 0.7 (Nunnally and Bernstein, 1994; Hair et al., 2019).

This confirms the internal consistency and reliability of the scale dimensions. To further verify the reliability and validity of the measurement model, confirmatory factor analysis (CFA) was employed. Convergent validity—the degree to which multiple items represent the same latent construct—was assessed using average variance extracted (AVE) and composite reliability (CR). As shown in Table 2, the AVE values for all the dimensions met the criteria (0.551–0.707), with risk-taking behavior (AVE = 0.707) and explicit performance (AVE = 0.674) performing best. The CR values ranged from 0.786 (power structure) to 0.878 (risk-taking behavior). The AVE and the CR exceeded the thresholds of 0.50 and 0.70, respectively, as recommended by Fornell and Larcker (1981), providing robust evidence for the convergent validity of the model. Discriminant validity analysis indicated that the constructs are significantly distinct from one another. As shown in Table 3, in all cases, the square root of the AVE for each construct (displayed on the diagonal) was greater than the interfactor correlation coefficient shown below it. Therefore, the study fully meets the fundamental criteria for establishing discriminant validity (Fornell and Larcker, 1981).

**Table 2:** Factor loadings and reliability of measurement items.

Items	Factor loading	*M*	*SD*	Cronbach’s alpha	AVE	CR
Role structure		4.04	0.70	0.805	0.579	0.805
	0.790					
	0.737					
	0.818					
Skill structure		3.93	0.85	0.825	0.621	0.831
	0.692					
	0.852					
	0.848					
Power structure		4.04	0.75	0.782	0.551	0.786
	0.709					
	0.727					
	0.830					
Risk-taking behavior		4.13	0.70	0.878	0.707	0.878
	0.800					
	0.858					
	0.865					
Decision-making behavior		4.10	0.74	0.788	0.568	0.797
	0.750					
	0.839					
	0.699					
Learning behavior		3.83	0.78	0.821	0.606	0.822
	0.802					
	0.838					
	0.821					
Developmental performance		4.24	0.69	0.813	0.597	0.816
	0.654					
	0.825					
	0.829					
Team cohesion		4.00	0.66	0.813	0.601	0.818
	0.792					
	0.835					
	0.814					
Explicit performance		4.03	0.79	0.859	0.674	0.861
	0.721					
	0.829					
	0.836					

**Table 3:** Pearson correlations and square roots of AVEs.

Latent variable	Role structure	Skill structure	Power structure	Risk-taking behavior	Decision-making behavior	Learning behavior	Developmental performance	Team cohesion	Explicit performance
Role structure	**0.760**								
Skill structure	0.323^**^	**0.788**							
Power structure	0.423^**^	0.316^**^	**0.742**						
Risk-taking behavior	0.355^**^	0.331^**^	0.349^**^	**0.840**					
Decision-making behavior	0.404^**^	0.351^**^	0.445^**^	0.353^**^	**0.753**				
Learning behavior	0.329^**^	0.145^*^	0.314^**^	0.250^**^	0.343^**^	**0.778**			
Developmental performance	0.390^**^	0.439^**^	0.429^**^	0.407^**^	0.429^**^	0.251^**^	**0.772**		
Team cohesion	0.195^**^	0.344^**^	0.305^**^	0.272^**^	0.179^**^	0.234^**^	0.249^**^	**0.775**	
Explicit performance	0.368^**^	0.425^**^	0.427^**^	0.335^**^	0.394^**^	0.343^**^	0.393^**^	0.410^**^	**0.820**

**Correlation is significant at the 0.01 level (2-tailed). The bold values on the diagonal represent the square root of the Average Variance Extracted(AVE) for each construct. These values are greater than the inter-factor correlation coefficients, which confirms the discriminant validity of the model.

### 4.3 Data normality assessment

Prior to constructing the structural equation model, the normality of the data distribution for the 250 observations was assessed using skewness and kurtosis statistics. The results indicated that the skewness values ranged from −1.568 to −0.391, and the kurtosis values ranged from −0.312 to 4.927. In accordance with the criteria proposed by Kline (2023), the data are considered to satisfy the assumption of univariate normality when the absolute value of the skewness is less than 2 and the absolute value of the kurtosis is less than 7. Thus, the sample data met the normality requirements for maximum likelihood estimation (MLE), justifying its use in the subsequent structural equation modeling analysis.

### 4.4 Hypothesis testing

Structural equation modeling (SEM) was subsequently performed to examine the theoretical relationships and hypothesized pathways between the latent constructs. The results demonstrated a good model fit: *χ*^2^ = 522.830, df = 308, *χ*^2^/df = 1.698, RMSEA = 0.053, CFI = 0.931, NFI = 0.848, TLI = 0.921, and AGFI = 0.839.

A total of 13 hypotheses were tested. At the team structure level, role structure showed a significant positive relationship with learning behavior (βRS → LB = 0.432, *p* < 0.001), supporting H1a. Additionally, role structure exerted a significant positive influence on decision-making behavior (βRS → DMB = 0.270, *p* = 0.003), supporting H1b. The path from skill structure to risk-taking behavior was significant (βSS → RTB = 0.245, *p* = 0.001), supporting H2b; however, the effect of skill structure on learning behavior was not significant (βSS → LB = 0.043, *p* = 0.585); thus, H2a was not supported. Power structure had significant positive effects on both decision-making behavior (βPS → DMB = 0.494, *p* < 0.001) and risk-taking behavior (βPS → DMB = 0.37, *p* < 0.001), supporting H3a and H3b. With respect to the relationship between team behavior and performance, learning behavior significantly and positively influenced cohesion (βLB → TC = 0.242, *p* = 0.002) and explicit performance (βLB → EP = 0.271, *p* < 0.001), supporting H4a and H4b. However, its effect on developmental performance was not significant (βLB → DP = 0.088, *p* = 0.204), failing to support H4c. Further results indicated that decision-making behavior had significant positive effects on cohesion (βDMB → TC = 0.221, *p* = 0.005), explicit performance (βDMB → EP = 0.436, *p* < 0.001), and developmental performance (βDMB → DP = 0.443, *p* < 0.001), supporting H5a, H5b, and H5c. Finally, the impact of risk-taking behavior on developmental performance was also significant (βRTB → DP = 0.276, *p* < 0.001), supporting H6 (Table 4).

**Table 4:** Path coefficients of the structural equation model (direct effects).

Direct effects Hypothesis	Path	Estimate (β)	S. E.	*T*	*p*-value	Results
H1a	RS → LB	0.432	0.103	4.843	***	Supported
H1b	SS → LB	0.043	0.069	0.546	0.585	Not supported
H2a	RS → DMB	0.27	0.098	2.958	0.003	Supported
H2b	SS → RTB	0.245	0.065	3.192	0.001	Supported
H3a	PS → DMB	0.494	0.1	4.993	***	Supported
H3b	PS → RTB	0.37	0.086	4.55	***	Supported
H4a	LB → TC	0.242	0.063	3.06	0.002	Supported
H4b	DMB → TC	0.221	0.067	2.8	0.005	Supported
H5a	LB → EP	0.271	0.077	3.78	***	Supported
H5b	DMB → EP	0.436	0.089	5.655	***	Supported
H6a	LB → DP	0.088	0.059	1.271	0.204	Not supported
H6b	DMB → DP	0.443	0.076	5.303	***	Supported
H6c	RTB → DP	0.276	0.063	3.845	***	Supported

The symbols *** indicate that the path coefficients are statistically significant at the *p* < 0.001 level.

**Figure 2:**
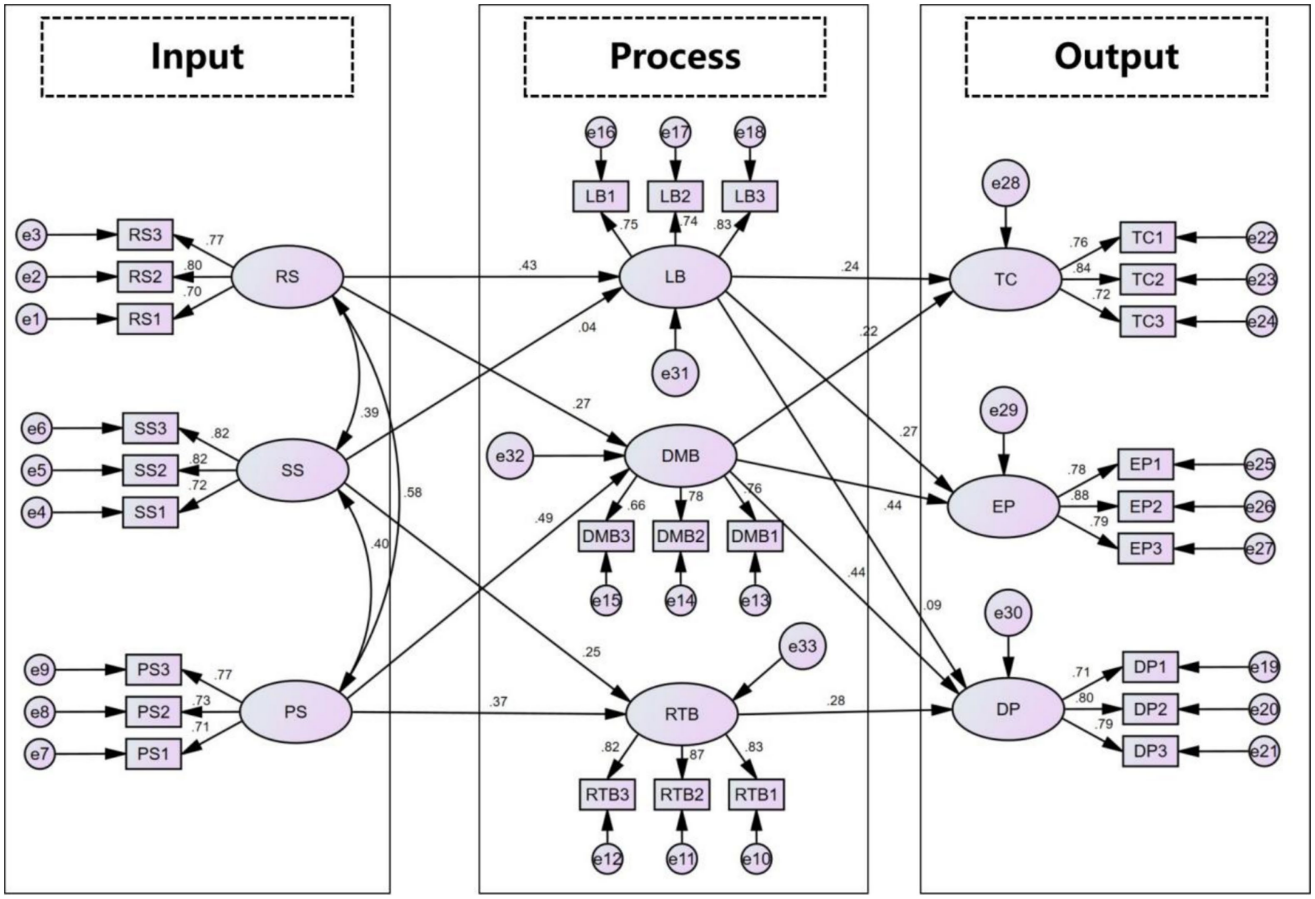
The resultant model of this research. Indirect effect (mediating): H7a: RS → LB → TC; H7b: RS → LB → EP; H8: PS → DMB → DP; H9: SS → RTB → DP.

This study further examined the mediating roles of team behaviors between team structure and performance. The results indicated that role structure had a significant indirect effect on team cohesion (*β* = 0.083; z = 2.90; *p* < 0.01) and explicit performance (*β* = 0.126; *z* = 3.52; *p* < 0.001) through learning behavior. This suggests that clear role differentiation not only fosters a proactive learning climate but also enhances team centripetal force and operational outcomes via learning activities, thereby supporting Hypotheses H7a and H7b. Second, power structure had a significant indirect effect on developmental performance through decision-making behavior (*β* = 0.199; *z* = 4.65; *p* < 0.001), supporting Hypothesis H8. These findings imply that a rational and scientific distribution of power facilitates synergistic efficiency during the decision-making process, which subsequently translates into the organization’s long-term growth potential. Finally, skill structure significantly promoted developmental performance via risk-taking behavior (*β* = 0.059, *z* = 2.78, *p* < 0.01), validating Hypothesis H9. This result indicates that a complementary skill structure strengthens the team’s adventurous spirit and willingness to engage in trial-and-error, thereby driving the firm’s adaptation and growth within uncertain environments (Figure 2 and Table 5).

**Table 5:** Indirect effects – mediation test.

Hypothesis	Path	Estimate (β)	*z*-value	*p*-value	95% CI Lower	95% CI Upper	Results
H7a	RS → LB → TC	0.083	2.90	0.004	0.027	0.139	Supported
H7b	RS → LB → EP	0.126	3.52	***	0.056	0.196	Supported
H8	PS → DMB → DP	0.199	4.65	***	0.114	0.284	Supported
H9	SS → RTB → DP	0.059	2.78	0.005	0.018	0.100	Supported

The symbols *** indicate that the path coefficients are statistically significant at the *p* < 0.001 level.

## 5 Discussion

### 5.1 Evidence and path mechanisms of the integrated IPO framework

This study systematically examines the applicability of the input-process-output (IPO) framework within the entrepreneurial teams of sports startups, focusing specifically on its explanatory power for team performance under conditions of high uncertainty and resource constraints. Within this framework, structural dimensions (role, skill, and power structures) serve as input variables, revealing how internal task division, skill heterogeneity, and power distribution shape subsequent team behaviors (Greer et al., 2017; Wallrich et al., 2024). Behavioral dimensions (learning, decision-making, and risk-taking behaviors) act as process variables, representing the team’s actual modes of action in daily operations, information integration, knowledge application, and responses to uncertainty (Wiese et al., 2021; Shepherd et al., 2023). The output dimensions encompass developmental/dynamic capability (DP), team cohesion (TC), and explicit performance (EP), covering multidimensional performance indicators that range from short-term execution to long-term adaptability (Mathieu et al., 2018; Grossman et al., 2021; Brock and Hitt, 2024).

The results demonstrate that structural elements significantly influence multidimensional performance through corresponding behavioral pathways. Specifically, a clear role structure facilitates the emergence of learning and decision-making behaviors, which in turn enhances team cohesion and explicit performance. This underscores the heuristic and constrained role of structure in fostering effective collaboration and high-quality execution. Furthermore, the decentralization of power and participatory governance bolster decision-making and risk-taking behaviors, subsequently improving both DP and EP, which indicates the core strategic importance of power allocation in the actions of entrepreneurial teams. The positive impact of skill structure on risk-taking behavior is also further supported, aligning with the resource-based view (RBV), which posits that skill diversity—as a critical resource—promotes exploratory and high-risk strategies (DeSarbo et al., 2007; Zahra et al., 2008; Cardella et al., 2021).

In addition, the study systematically validates the applicability of the IPO framework within sports startup teams, particularly from the perspective of resilience-relevant behaviors. It confirms the validity of pathways through which structural dimensions influence performance outputs via behavioral processes. The findings indicate that structural elements are transmitted to multidimensional performance through specific behavioral mechanisms. Overall, the study supports the IPO framework as a comprehensive model for explaining the performance of sports entrepreneurial teams, revealing how structural elements collectively drive multidimensional outputs through designated behavioral processes.

Furthermore, the path coefficient of RS → DMB is 0.270 (*p* = 0.003), representing a moderate effect size that is statistically significant. Within an integrated IPO (Input-Process-Output) framework, individual path coefficients are typically smaller than those in bivariate analyses, as explanatory power is distributed across multiple mediating pathways (Marks et al., 2001). This finding aligns with existing research on role clarity enhancing decision efficiency (Eisenhardt, 1989; Hoeber and Hoeber, 2012). However, the moderate magnitude of the effect size also reflects the distinctive nature of the sports entrepreneurship context: role structures must simultaneously serve technical-entrepreneurial objectives and sports-domain requirements, and the complexity of this cross-domain collaboration imposes boundary conditions on the direct influence of role clarity on decision-making behavior.

Moreover, the mediation architecture of our model indicates that role structure (RS) influences performance not only through decision-making behavior (DMB), but also via indirect effects through learning behavior (LB) and risk-taking behavior (RTB). These pathways collectively amplify the total impact of structural dimensions on outcome variables such as developmental performance (DP) and team cohesion (TC). From a practical standpoint, this finding suggests that in the organizational design of sports startup teams, attention should be given simultaneously to role clarification and the synergistic intervention of multiple behavioral pathways, thereby enhancing the comprehensive explanatory power for performance outcomes.

### 5.2 Mechanisms and heterogeneity of the structure–behavior–performance pathway in sports startups

Role structure (RS) has a significant positive effect on both learning and decision-making behaviors. This finding is highly consistent with classical team effectiveness theories, which posit that a clear definition of roles and responsibilities constitutes the structural foundation for effective collaboration and collective decision-making (Cohen and Bailey, 1997). This study further reveals the significant heuristic and constraining effects of structural elements on behavioral processes. Specifically, while a clear role structure demands a distinct division of labor, it simultaneously enhances team cohesion (TC) and explicit performance (EP) by facilitating the synergy between learning behavior (LB) and decision-making behavior (DMB) (Chen et al., 2020; Grossman et al., 2021; Wiese et al., 2021; Shepherd et al., 2023). In the high-uncertainty environment of sports startups, precise role and responsibility definitions are prerequisites for efficient collaboration and execution. Moreover, this study emphasizes that the marginal effect of role structure on process shaping is more pronounced in resource-constrained contexts, where role clarity reduces information search costs and improves cross-functional coordination efficiency.

The study also reveals that decentralization and increased participation in power structure (PS) significantly bolster the implementation of decision-making and risk-taking behaviors, which in turn influence the formation of developmental/dynamic capability (DP) and team cohesion. Thus, power allocation emerges as a critical strategic variable influencing team action pathways. Particularly in the early stages of a venture, decentralized governance promotes rapid iteration and enhances the team’s consensus and coping capacity when facing uncertain challenges. Furthermore, the positive impact of skill structure (SS) on risk-taking behavior (RTB) was confirmed, providing further empirical support for the application of the resource-based view (RBV) in the context of sports startups (Cardella et al., 2021). As a core resource, skill diversity facilitates the pursuit of exploratory and high-risk strategies, providing the necessary resource resilience for a team’s survival and growth within uncertain environments (Edmondson, 1999; DeSarbo et al., 2007).

Learning behavior significantly positively influences both team cohesion and explicit performance. These findings align with organizational learning theory and existing research on team learning, which posits that by continuously acquiring, sharing, and applying knowledge, teams can enhance collaboration, trust, and identity among members, thereby increasing team cohesion while improving short-term operational performance through process optimization and capability accumulation (Argote, 2012). Furthermore, decision-making behavior (DMB) and risk-taking behavior (RTB) significantly mediate the relationship between power structure (PS) and developmental performance (DP). This reveals the direct driving force of action-oriented strategic behavioral pathways in the formation of long-term dynamic capabilities. These findings emphasize that in pursuit of long-term innovation and competitive advantage, organizations should harmonize power structure design, decision-making tempo, and risk appetite to ensure that strategic actions can consistently accumulate over time and translate into dynamic capabilities (Horvatinovic et al., 2023). The positive impact of skill structure (SS) on risk-taking behavior (RTB) and its relationship with resource integration and exploratory decision-making are consistent with recent studies suggesting that skill diversity enhances competitive advantage in uncertain environments (DeSarbo et al., 2007; Zahra et al., 2008; Cardella et al., 2021). Notably, some research hypotheses were not supported. The effect of skill structure on learning behavior was not significant (*β* = 0.088, *p* = 0.204). This finding creates a theoretical tension with the traditional view in team learning theory that “skill diversity facilitates learning behavior”. The results suggest that in the startup phase of sports ventures, a highly heterogeneous skill structure does not necessarily translate into more frequent or effective team learning behaviors. This phenomenon may stem from the severe resource and time constraints faced by entrepreneurial teams during their inception. In the early stages of sports startups, while high skill heterogeneity expands the potential knowledge base, it also significantly increases cognitive conflict and knowledge integration costs. Differences in professional terminology, problem conceptualization, and operational logic among members of diverse backgrounds may hinder a team’s ability to form efficient, integrated learning mechanisms within a limited timeframe. Additionally, the direct effect of learning behavior on developmental performance was non-significant ($\beta = 0.088, *p* = 0.204$). This finding indicates that while learning behavior enhances incremental outcomes such as team cohesion and explicit performance, it does not directly impact “developmental performance,” which reflects long-term strategic innovation and environmental adaptability. This may be because, in new ventures, the formation of developmental performance relies more on strategic, outward-looking action choices than purely inward-looking knowledge absorption and sharing processes. Compared with continuous learning and incremental improvement, pivotal decision-making and high-risk strategic actions (e.g., major investments, business model innovation, and market entry) have a more direct and significant impact on resource reconfiguration and opportunity capture, thereby driving the formation and accumulation of dynamic capabilities to a greater extent. Taken together, these mechanisms support an integrated framework in which structure drives behavior and behavior drives performance. This study validates the applicability of core propositions from classical theories within the context of sports entrepreneurship and reveals how contextual factors, such as resource constraints and uncertainty, strengthen path intensity and stability through contextual mediators such as resilience-relevant behaviors.

### 5.3 Significance, limitations, and future directions

This study systematically applies the input–process–output (IPO) framework to teams in sports startups and embeds resilience-relevant behaviors into the structure–process–outcome chain. It reveals how resilience-relevant behaviors the influence of structure on processes, strengthens the intensity of mediating effects, and enhances the sustainability of performance under conditions of high uncertainty. Theoretically, the findings extend the boundaries of cross-disciplinary research between team effectiveness and organizational resilience, highlighting the importance of balancing outward-looking, proactive actions with internal learning in resource-constrained entrepreneurial environments. Methodologically, the study emphasizes a comprehensive assessment where both direct and indirect effects coexist, suggesting that future research in complex contexts should employ techniques such as multigroup structural equation modeling (SEM), latent variable interactions, and situational variables (e.g., market maturity, resource munificence, and team stages) to uncover pathway heterogeneity and boundary conditions.

Despite these contributions, several limitations should be acknowledged. First, the use of convenience sampling in two Chinese regions (Quanzhou and Zhenjiang) may limit the generalizability of the findings (McEwan, 2020; Emerson, 2021). Although the selected regions represent active sports entrepreneurship ecosystems, potential selection bias cannot be fully excluded (Infante-Rivard and Cusson, 2018). Second, the cultural and institutional context of China may shape power distribution norms and collective behaviors differently from Western contexts (Klett and Arnulf, 2020; Huang et al., 2019).

With respect to theoretical boundaries and contextual applicability, while this framework is supported within the sports sector, contextual variables—such as industry maturity, market norms, and organizational life cycles—may cause conditional variances in the intensity of pathways and the significance of mediating effects. Consequently, future research should test the robustness of the IPO framework through cross-industry horizontal designs and longitudinal life-cycle designs. Expanding the scope to include more sports subsectors and geographical regions will help evaluate the cross-industry generalizability and adaptability of the framework across different developmental stages.

In terms of methodology, future studies should prioritize longitudinal research designs to capture the dynamic processes of structural adjustment, behavioral evolution, and performance changes. Integrating longitudinal experiments or quasi experimental designs could further strengthen causal inference. Furthermore, the direct and indirect effects can be triangulated through multiple data sources (e.g., surveys, interviews, behavioral logs, and objective performance metrics). Future research should also explore the equilibrium point between learning mechanisms and action-oriented mechanisms in contexts where resource constraints and time pressures coexist, thereby clarifying the core mechanisms that should be prioritized at different organizational stages.

## 6 Conclusion and recommendations

### 6.1 Conclusion

On the basis of the input-process-output (IPO) framework, this study systematically examines the transmission mechanisms of structural elements (role, skill, and power structures) through behavioral processes (learning, decision-making, and risk-taking behaviors) on multidimensional performance outputs (developmental performance/dynamic capability, team cohesion, and explicit performance) among sports startup teams. The results indicate that structural elements significantly influence multidimensional performance through corresponding behavioral pathways, confirming the validity of the “structure–behavior–performance” integrated path in the context of sports entrepreneurship. Furthermore, the positive impact of skill structure on risk-taking behavior received empirical support, aligning with the core tenets of the resource-based view (RBV), which posits that skill diversity—as a critical resource—drives the implementation of exploratory and high-risk strategies. By strengthening mediating effects, resilience-relevant behaviors enhances the stimulus effect of structure on behavioral processes and improves the formation and stability of short-term performance and long-term dynamic capabilities. These findings extend the application boundaries of the traditional IPO framework in high-uncertainty entrepreneurial contexts, particularly highlighting the critical role of integrating structural design with behavioral responses to achieve superior performance in sports startups. Overall, the study supports the IPO framework as a comprehensive model for explaining team performance in sports entrepreneurship, revealing how structural elements collectively drive multidimensional outputs through specific behavioral processes and clarifying the unique value of resilience-relevant behaviors in enhancing pathway efficacy.

### 6.2 Recommendations

Prioritize Structural Design: Within the governance framework of sports startups, ensuring a clear role structure (RS) with well-defined responsibility boundaries is essential for enhancing cross-functional collaboration, information flow, and execution while reducing information search costs and coordination friction, thereby strengthening the rationality of risk-taking. Second, enhancing the diversity and complementarity of the skill structure (SS) can improve resource elasticity and the ability to cope with uncertainty, supporting exploratory and innovative collaboration. Furthermore, promoting moderate decentralization of the power structure (PS) and implementing participatory decision-making can improve decision quality, shorten cycles, and create an arena for learning opportunities and knowledge sharing. In practice, strengthening skill structure may involve developing digital and AI-related competencies to enhance data-driven decision-making in sports ventures (Paul et al., 2022; Vec et al., 2024). Optimizing power structure could include leadership training programs that promote participative decision-making while maintaining strategic clarity (Lacerenza et al., 2017; Wang et al., 2022).

Focus on Efficient Synergy of Learning and Decision-making: Learning behavior (LB) should be institutionalized into daily operations through regular knowledge sharing, cross-team workshops, and rapid-iteration project reviews to form a continuous learning loop and accumulate organizational memory. Enhancing decision-making behavior (DMB) relies on systematic information integration and consensus-building; thus, a rapid and high-quality collaborative decision-making process must be established to ensure efficient alignment between structure and learning mechanisms. With respect to risk-taking behavior (RTB), a controllable experimental environment and fault-tolerance mechanism should be established to advance exploratory actions within manageable risk boundaries, promoting a balance between innovation and safety.

Systematically Cultivate Team resilience-relevant behaviors. In high-uncertainty environments, teams should foster psychological safety and a learning culture while strengthening organizational elasticity training for rapidly changing environments. The improvement in resilience-relevant behaviors not only amplifies the effects of the structure–behavior pathway but also enhances its stability and sustainability across different contexts, providing stronger adaptability for long-term performance and the formation of dynamic capabilities.

Obtain Robust Instrumental Support from External Stakeholders: Governments, industry associations, and incubators should provide strong instrumental support, such as entrepreneurial education, multidisciplinary collaborative platforms, and resource-matching mechanisms. These efforts can facilitate structural optimization and the cultivation of a learning culture in sports startup teams, enhancing industry innovation and overall performance. For sports startups, policy guidance should help form a closed-loop governance that integrates structural design, learning mechanisms, and resilience cultivation to transform institutional support into actual performance enhancement and competitive advantage.

In summary, structural design provides an efficient operational framework for behavioral processes through the synergistic optimization of RS, SS, and PS, while the mediating mechanisms of LB, DMB, and RTB facilitate the transformation into long-term performance and dynamic capabilities. Future research and practice should deepen the examination of pathway heterogeneity across horizontal and vertical dimensions, expand mechanism networks, and embed policy tools into corporate governance and ecosystem construction to enhance the overall innovation and competitiveness of the sports entrepreneurship ecosystem.

## Data Availability

The original contributions presented in the study are included in the article/supplementary material, further inquiries can be directed to the corresponding author.
